# Reliability and validity of the simplified Chinese version of the Musculoskeletal Health Questionnaire (MSK-HQ-C) in patients with non-specific chronic neck pain: a cross-cultural adaptation and validation study

**DOI:** 10.1186/s40359-026-04288-w

**Published:** 2026-03-13

**Authors:** Wenbo Diao, Peng Wang, Pengyi Wei, Xiaosong Huang, Lulu Jia

**Affiliations:** 1 Department of Orthopedics, Zhoukou Orthopedic Hospital, Zhoukou City, Henan Province People’s Republic of China; 2 The First Department of Orthopedics and Traumatology, The First Affiliated Hospital of Hebei University of Chinese Medicine, Zhongshan Dong Rd. 389, Changan District, Shijiazhuang City, Hebei Province People’s Republic of China; 3 Sports Medical Rehabilitation Room, Hebei Institute of Sport Science, Shijiazhuang City, Hebei Province People’s Republic of China; 4 The First Department of Orthopedics and Traumatology, Luanping County Hospital of Traditional Chinese Medicine, Luanping County, Baojian Rd. 57, Luanping Town, Luanping County, Chengde City, Hebei Province People’s Republic of China; 5 Department of Anesthesiology, Shijiazhuang People’s Hospital, Fangbei Rd. 9, Changan District, Shijiazhuang City, Hebei Province People’s Republic of China

**Keywords:** Musculoskeletal Health Questionnaire, Cross-cultural adaptation, Validity, Reliability, Non-specific chronic neck pain, Quality of life, Simplified Chinese

## Abstract

**Background:**

The Musculoskeletal Health Questionnaire (MSK-HQ) is a 14-item instrument designed to holistically assess musculoskeletal health across domains of pain, physical function, sleep, emotional well-being, and social participation. This study aimed to translate and cross-culturally adapt MSK-HQ into a Simplified Chinese version (MSK-HQ-C), and evaluate the reliability and validity of MSK-HQ-C in patients with non-specific chronic neck pain.

**Methods:**

A total of 150 participants were included in this study. Internal consistency was estimated according to Cronbach’s alpha. Test-retest reliability was assessed by Intra-class correlation coefficient (ICC) based on completions from all participants at a 7–14 day interval. Construct validity was analyzed by correlations between MSK-HQ-C and the EuroQol-5 Dimensions-5 Levels (EQ-5D-5 L), Copenhagen Neck Function Disability Scale (CNFDS) as well as the short form (36) health survey (SF-36). The structural, convergent, and discriminant validity of the questionnaire were assessed using factor analysis and correlation analyses.

**Results:**

The original version of the MSK-HQ was cross-culturally adapted and translated into Simplified Chinese following the guidelines by Beaton et al. MSK-HQ-C was indicated to have excellent reliability (Cronbach’s alpha = 0.922, ICC = 0.822). Moderate to substantial correlations between MSK-HQ-C and EQ-5D-Total (*r*= .648, *p* < .001), EQ-5D-VAS (*r*= .707, *p* < .001), CNFDS (*r* = − .759, *p* < .001), as well as physical function (*r*= .594, *p* < .001), role-physical (*r*= .407, *p* < .001), bodily pain (*r*= .512, *p* < .001) and general health (*r*= .496, *p* < .001) subscales of SF-36 were observed. An exploratory factor analysis revealed that the 2-factor loading explained 74.206% of the variance (KMO = 0.919; Bartlett’s test of sphericity: χ² = 1855.421, *p* < .001).

**Conclusion:**

MSK-HQ-C was demonstrated to have acceptable reliability and validity in patients with non-specific chronic neck pain, which could be recommended for patients in Chinese mainland.

**Supplementary Information:**

The online version contains supplementary material available at 10.1186/s40359-026-04288-w.

## Introduction

Musculoskeletal (MSK) disorders, including chronic pain, osteoarthritis, and inflammatory conditions, represent a leading cause of global disability, profoundly impacting physical function, psychological well-being, and quality of life [1–3]. In China, the burden of MSK conditions is substantial, with an estimated 20–30% of adults reporting chronic musculoskeletal pain, and MSK has been the top causes of years lived with disability [4]. However, culturally adapted tools to assess these outcomes remain limited. Patient-reported outcome measures (PROMs) are critical for evaluating symptom severity, treatment efficacy, and healthcare needs in both clinical and research settings. However, their validity hinges on linguistic accuracy and cultural relevance for target populations.

Existing Simplified Chinese PROMs for MSK conditions, such as the Oswestry Disability Index and DASH Questionnaire, focus narrowly on region-specific disabilities (e.g., low back pain, upper limb dysfunction) and lack the MSK’s multidimensional scope [5, 6]. Furthermore, direct translations of PROMs risk misinterpretation due to linguistic nuances, cultural perceptions of pain, and differing healthcare-seeking behaviors. Hence, before PROMs are used in different cultural backgrounds, it is necessary to not only translate questionnaires well linguistically, but also maintain the content validity of the instrument to avoid bias due to cultural variety [7–9].

The Musculoskeletal Health Questionnaire (MSK-HQ), developed by Hill et al. (2014), is a 14-item instrument designed to holistically assess MSK health across domains of pain, physical function, sleep, emotional well-being, and social participation [10]. Its brevity, psychometric robustness, and alignment with international standards for MSK care (e.g., WHO’s Rehabilitation 2030 Initiative) have led to its cross-cultural adaptation in multiple languages, including Arabic, Danish, European Portuguese, German, Hungary, Marathi, and Norwegian [11–17]. However, a validated Simplified Chinese version does not yet exist, hindering its application in mainland China’s diverse clinical and research contexts.

This study aims to (1) cross-culturally adapt the MSK-HQ into Simplified Chinese following international guidelines for PROM translation and (2) evaluate its psychometric properties, including internal consistency, test-retest reliability, and construct validity. By addressing this gap, the Simplified Chinese MSK-HQ will facilitate standardized assessment of MSK health in Chinese-speaking populations, enabling comparative research and culturally informed care.

## Methods

### Translation and cross-cultural adaptation

The translation and cultural adaptation of the MSK-HQ followed internationally recognized guidelines for PROMs [18, 19]. The process included:

1. Forward Translation: Two bilingual translators independently translated the original English MSK-HQ into Simplified Chinese.
2. Synthesis: A committee (including translators, a rheumatologist, and a methodologist) reconciled discrepancies to produce a consensus version.
3. Back Translation: Two additional translators, blinded to the original questionnaire, translated the Chinese draft back into English.
4. Expert Committee: The committee compared back-translations with the original MSK-HQ to resolve semantic, idiomatic, and conceptual inconsistencies.
5. Pilot Testing: The pre-final version was administered to 15 patients with neck pain to assess comprehension and relevance. Feedback informed minor revisions. The demographic and clinical characteristics of the 15 patients participating in the pilot test are summarized in Table S1.

### Study population

From September 2021 to November 2023, participants were recruited via consecutive sampling from the outpatient clinics of the involved hospitals during the study period. Inclusion criteria: (1) age ≥ 18 years, (2) self-reported neck pain lasting ≥ 3 months, and (3) fluency in Simplified Chinese. Exclusion criteria: (1) cognitive impairment, (2) severe psychiatric illness, (3) recent neck surgery (< 6 months), (4) unstable condition of illness during measurement gap, (5) other uncontrolled systemic diseases, such as diabetes mellitus, malignant tumor or symptomatic hepatitis. Participants who met al.l inclusion criteria and did not meet any of the exclusion criteria were recruited to participate our study. Ethical approval was obtained from Ethics Committee of authors’ hospital, and written informed consent was acquired from all participants. The sample size of patients should also meet the standard raised by Terwee et al. [20], which indicated at least 50 patients should be included in the study to test floor or ceiling effects, reliability and validity of questionnaire.

### Data collection

Participants completed the following questionnaires at baseline (T1):

- Simplified Chinese MSK-HQ: 14 items scored 0–4 (total range: 0–56; higher scores indicate better musculoskeletal health).
- Criterion Measures:
- Validated simplified Chinese version of EuroQol-5 Dimensions-5 Levels (EQ-5D-5 L): Assessed health-related quality of life for convergent validity.
- Copenhagen Neck Function Disability Scale (CNFDS): Measured neck-specific disability.
- Short form (36) health survey (SF-36): General health status tool for discriminant validity.

All of the above instruments have been translated into Simplified Chinese and proven with good reliability and validity [21–23].

All participants were asked to fill MSK-HQ-C again 7–14 days after the first test to assess test-retest reliability. This interval was chosen to minimize the risk of recall bias (which can occur with shorter intervals) while reducing the likelihood of true clinical change (which is more likely with longer intervals), reflecting typical outpatient follow-up schedules in our clinical setting.

### Psychometric evaluation

Participants were asked the difficulties encountered when they fulfilled MSK-HQ-C. Score distribution of each item was analyzed. Ceiling and floor effects were considered to be present if more than 15% of patients achieved lowest (0) or highest (56) possible total scores [24]. 

#### Reliability

Cronbach’s α ≥ 0.70, 0.80 and 0.90 were considered acceptable, good and excellent internal consistency, respectively [20]. Intraclass correlation coefficient (ICC), deriving from a two-way random-effects model for absolute agreement (ICC(2,1)), was calculated for scores between T1 and T2 to assess test-retest reliability, of which higher than 0.8 and 0.9 were considered as good and excellent reliability, respectively [25]. Bland-Altman plots was used to evaluate systematic bias between the two measures [26].

#### Validity

Validity tests for MSK-HQ-C were evaluated regarding content validity and construct validity.

For content validity, one physical therapist and three orthopaedists analyzed if the content in each item could well measure and represent patients state of disease. Each expert rated the relevance of individual items to their corresponding content dimensions using a 4-point scale (1 = not relevant, 2 = weakly relevant, 3 = strongly relevant, 4 = very relevant). The item-level content validity index (I-CVI) for each item was calculated as the ratio of experts who gave a rating of 3 or 4 to the total number of experts. The scale-level content validity index (S-CVI/Ave, average) was defined as the percentage of items that received a rating of 3 or 4 from all experts. When the number of experts is ≤ 5, an I-CVI of 1.00 is considered to indicate good content validity for the corresponding item.

For construct validity, Pearson correlation coefficients (r) between MSK-HQ-C and EQ-5D-5 L, CNFDS, as well as subscales of SF-36 were calculated. Then the construct validity for MSK-HQ-C was judged as poor (*r*=-.2 ~ .2), fair (*r* = .2 ~ .4/-0.4 ~ -0.2), moderate (*r* = .4 ~ .6/-0.6 ~ -0.4), substantial (*r* = .6 ~ .8/-0.8 ~ -0.6), or almost perfect(*r* = .8 ~ 1.0/-1.0 ~ -0.8), according to r value [27]. We hypothesized that score of MSK-HQ-C should correlate well with EQ-5D-5 L, CNFDS and subscales of physical component of SF-36 (convergent validity), but poorly with mental component of SF-36 (divergent or discriminant validity).

Factorial validity is used to establish the factor structure of the scale [28]. Exploratory Factor Analysis (EFA) was conducted on a randomly split half-sample (*n* = 75) using principal axis factoring with Promax rotation (Kaiser-Meyer-Olkin [KMO] ≥ 0.60; Bartlett’s test *p* < .05).

All statistical analyses were conducted using the IBM SPSS Statistics for Windows, Version 26.0. The critical values for significance were set at *p* < .05.

## Results

### Participants

From September 2021 to November 2023, a total of 191 patients were invited to participate in the study, and 150 patients (78.5%) agreed and were included in this study finally. All patients completed two rounds of instruments without withdrawn cases. Table 1 listed the detailed demographic and clinical characteristics of participants.

**Table 1:** Demographic and clinical characteristics of participants

Characteristics	Range	Mean ± SD or *n* (%)
Age (years)	21–72	45.3 ± 11.6
Gender	Total = 150	
Female		71 (47.3%)
Male		79 (52.7%))
Education		
Elementary		2 (1.3%)
Mid school		24 (16.0%)
High school		31 (20.7%)
Graduate school		93 (62.0%)

### Translation and cross-cultural adaptation process

The translation process resolved key linguistic challenges. For instance, the English terms “aches” and “pains”, both broadly translated as 疼痛 in previous studies, were differentiated as 酸痛 (dull ache/soreness) and 疼痛 (sharp/intense pain) to capture distinct nuances. The item “washing” was adapted to 洗漱 (washing/grooming), and “daily routine” was rephrased as 日常生活安排 (daily life arrangements) to align with colloquial Mandarin. Intensity descriptors like “extremely” and “very” were calibrated to 极其 and 非常, reflecting culturally appropriate gradations. Pilot testing confirmed the pre-final version’s clarity, with none of participants reporting any difficulties in comprehension.

### Acceptability and score distribution

The Simplified Chinese MSK-HQ demonstrated excellent acceptability, with no missing responses exceeding 2% for any item. Total scores ranged from 0 to 55 (mean = 30.35, SD = 10.77). Absolute values of all 4 instruments were listed in Table 2. No floor (0.67%) or ceiling effects (0%) were observed (< 15% at score extremes) [24].

**Table 2:** Absolute values of all scores

Scales	Mean ± SD	Minimum	Median	Maximum
EQ-5D				
Total score	0.354 ± 0.210	− 0.111	0.339	1.000
Health status (VAS)	45.2 ± 17.7	2	42	95
CNFDS	16.2 ± 5.5	5	16	28
SF-36				
Physical functioning	42.8 ± 17.6	0	40	85
Role-physical	39.0 ± 25.3	0	25	100
Bodily pain	35.8 ± 14.8	0	41	84
General health	40.4 ± 19.1	0	40	100
Vitality	38.9 ± 18.4	0	40	90
Social functioning	43.5 ± 21.1	0	37.5	100
Role-emotional	43.6 ± 21.1	0	33.3	100
Mental health	41.7 ± 17.3	4	40	92

*SD* Standard deviation, *EQ-5D* EuroQol-5 dimensions, *CNFDS* Copenhagen Neck Functional Disability Scale, *SF-3*6 Short form 36

### Reliability

**Fig. 1:**
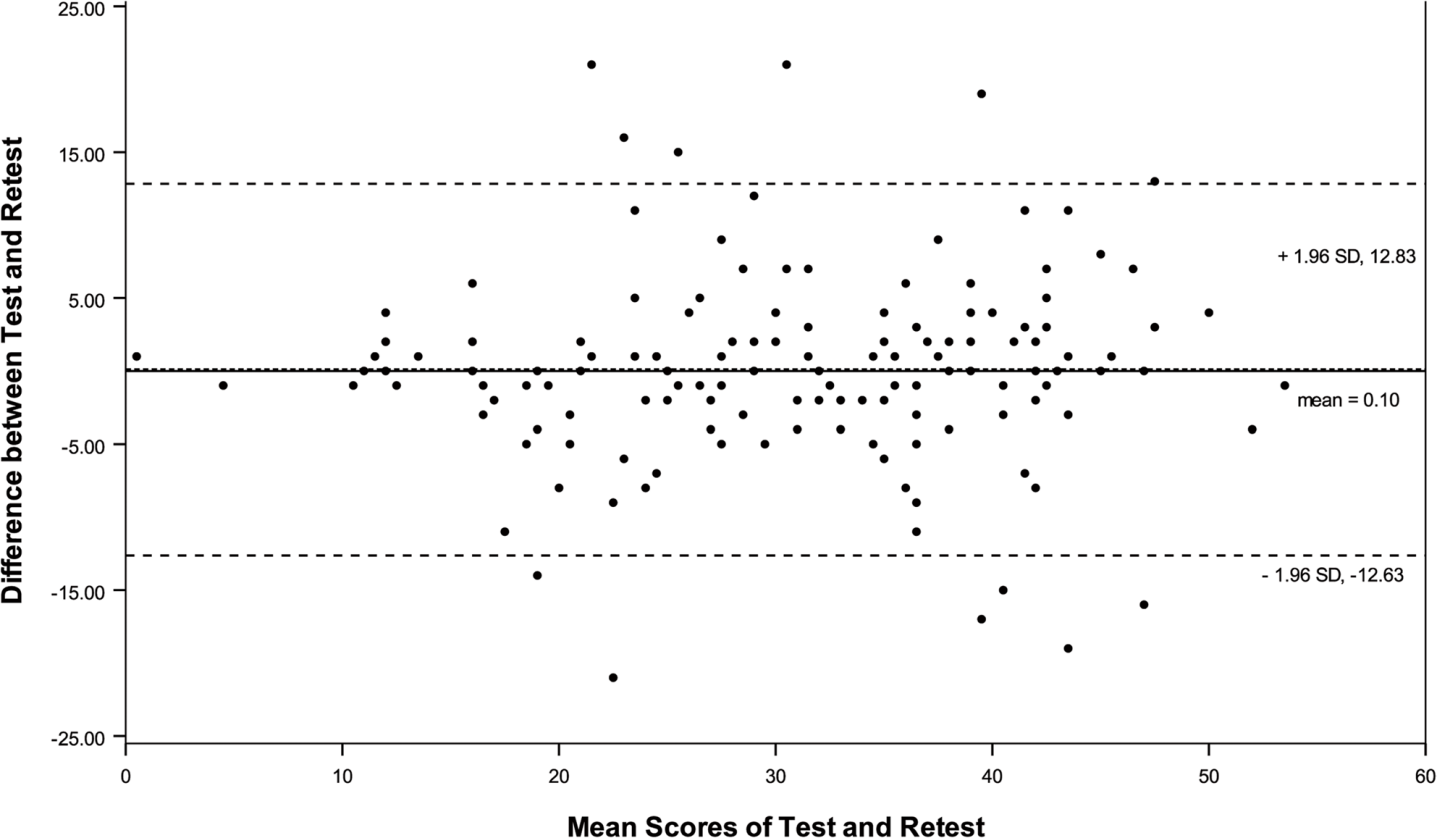
The Bland-Altman plot for test-retest agreement of MSK-HQ. The differences between scores for MSK-HQ from test and retest were plotted against the mean of the test and retest. The line indicates mean difference value of the two sessions and the 95% (mean ± 1.96 standard deviation) limits of agreement

Internal consistency assessed by Cronbach’s α for the total scale was 0.922, exceeding the threshold of 0.70. Mean scores of MSK-HQ-C were comparable between the two rounds of test. The ICC between baseline and follow-up scores was 0.822 (95% CI [0.762, 0.868]), indicating excellent stability. Elimination of one item in all 14 questions did not result in a value of < 0.90. All items correlated with the total score of > 0.471 (Table 3). Bland-Altman analysis revealed no systematic bias (Fig. 1).

**Table 3:** Item-level statistics and internal consistency of the MSK-HQ-C at baseline (T1)

Item	Mean ± SD	Item-total correlation	Cronbach’s α if item deleted
1	2.26 ± 1.13	0.561	0.919
2	2.25 ± 1.12	0.587	0.918
3	2.16 ± 1.02	0.750	0.913
4	2.19 ± 1.03	0.773	0.912
5	2.08 ± 0.97	0.714	0.914
6	2.13 ± 0.99	0.749	0.913
7	2.24 ± 1.12	0.606	0.918
8	2.09 ± 1.06	0.580	0.919
9	2.15 ± 1.10	0.625	0.917
10	2.12 ± 1.29	0.471	0.924
11	2.26 ± 1.16	0.626	0.917
12	2.05 ± 1.09	0.784	0.912
13	2.10 ± 0.95	0.777	0.913
14	2.27 ± 1.22	0.592	0.919

### Validity

According to the assessment of physical therapist and orthopaedists, 13 out of 14 items of MSK-HQ-C achieved an I-CVI of 1.0, indicating that all 4 experts agreed that these items were moderately or highly relevant to the assessment of musculoskeletal health in patients with non-specific chronic neck pain. Only item 12 (“Understanding of your condition and any current treatment”) had an I-CVI of 0.75, corresponding to 3 experts rating it as 3 or 4 and 1 expert rating it as 2 (weakly relevant). S-CVI/Ave, calculated as the mean of all item-level I-CVIs, was (13 × 1.00 + 0.75)/14 = 0.982. According to the criterion that S-CVI/Ave ≥ 0.90 indicates excellent content validity, the scale achieved outstanding overall content validity.

Table 4 lists the correlation coefficients of MSK-HQ-C with CNFDS, EQ-5D and SF-36 subscales, which proved good construct convergent and discriminant validity of MSK-HQ-C. The total score of MSK-HQ-C showed substantial correlation with the CNFDS (*r* = − .759, *p* < .001), EQ-5D (*r* = .648, *p* < .001) and EQ-VAS (*r* = .707, *p* < .001), and moderate correlation with physical function (*r*= .594, *p* < .001), role-physical (*r*= .470, *p* < .001), bodily pain (*r*= .512, *p* < .001) and general health (*r*= .496, *p* < .001) subscales of SF-36 (Table 4). Besides, MSK-HQ-C correlated fairly with vitality (*r*= .268, *p* = .001), social function (*r*= .205, *p* = .012) and mental health (*r*= .349, *p* < .001) subscales of SF-36, and was not relevant to role-emotional (*r*= .120, *p* = .145) subscale of SF-36, which consistently matches our hypothesis.

**Table 4:** Construct validity of MSK-HQ-C

Scales	Correlation coefficient *r*_*p*_^a^	*p* value
EQ-5D		
Total score	0.648^**^	< 0.001
Health status (VAS)	0.707^**^	< 0.001
CNFDS	− 0.759^**^	< 0.001
SF-36 subscales		
Physical Function	0.594^**^	< 0.001
Role-Physical	0.470^**^	< 0.001
Bodily Pain	0.512^**^	< 0.001
General Health	0.496^**^	< 0.001
Vitality	0.268^**^	0.001
Social Function	0.205^*^	0.012
Role-Emotional	0.120	0.145
Mental Health	0.349^**^	< 0.001

The sample size for the analysis of construct validity was 150

MSK-HQ-C: Simplified Chinese version of Musculoskeletal Health Questionnaire; EQ-5D: EuroQol-5 dimensions; CNFDS: Copenhagen Neck Functional Disability Scale; SF-36: short form 36

^*^Correlation is significant at the 0.05 level (two-tailed)

^**^Correlation is significant at the 0.01 level (two-tailed)

^a^ Calculated by the Pearson’s correlation coefficient (*r*_*p*_) of the MSK-HQ-C with EQ-5D, CNFDS and SF-36

**Fig. 2:**
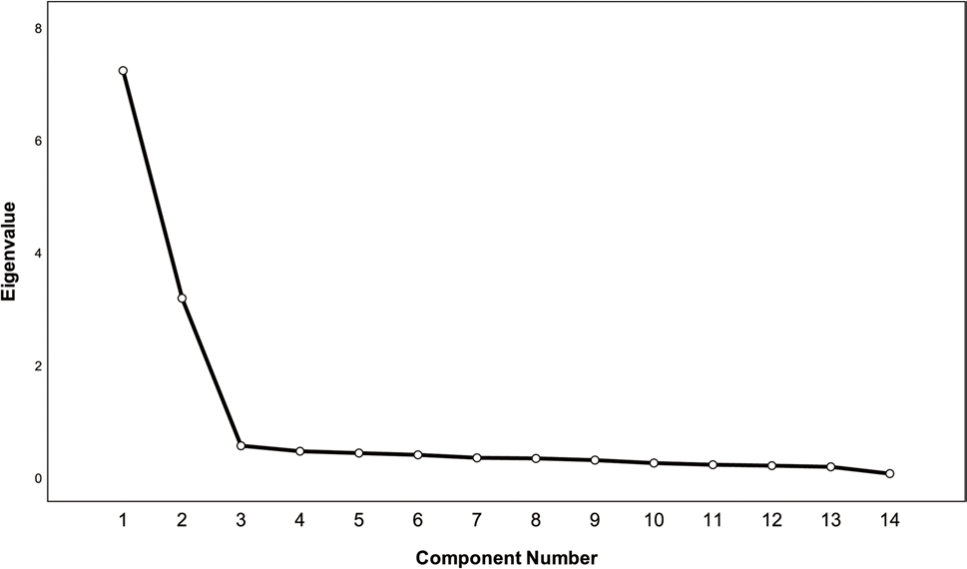
Scree plot of eigenvalues from the 14-item MSK-HQ-C

EFA showed a 2-factor solution explained 74.206% of the variance (KMO = 0.919; Bartlett’s test of sphericity: χ² = 1855.421, *p* < .001), in which items 1–6, 12, and 14 grouping into domains of physical function, and item 7–11, and 13 grouping into emotional/social impact. (Table 5; Fig. 2) These exploratory findings should be interpreted with caution given the modest split-sample size. Internal consistency, assessed by Cronbach’s α, was 0.940 for physical function and 0.921 for social participation; the test-retest reliability, measured by ICC, was 0.829 (95% CI [0.764, 0.876]) and 0.871 (95% CI [0.822, 0.907]), respectively. Table S2 lists the correlation coefficients of Physical Function and Emotional/Social Impact Factors of MSK-HQ-C with CNFDS, EQ-5D and SF-36 subscales.

**Table 5:** Factor loading values for all items of MSK-HQ-C

MSK-1	1	2
	0.922	
MSK-2	0.923	
MSK-3	0.812	
MSK-4	0.806	
MSK-5	0.762	
MSK-6	0.749	
MSK-7		0.885
MSK-8		0.895
MSK-9		0.829
MSK-10		0.790
MSK-11		0.842
MSK-12	0.705	
MSK-13		0.716
MSK-14	0.842	

## Discussion

This study cross-culturally adapted the MSK-HQ into Simplified Chinese and validated its psychometric properties in a population with chronic neck pain. The results support its reliability and validity for assessing musculoskeletal health in Mandarin-speaking clinical and research contexts.

While region-specific tools like the NDI and CNFDS are excellent for assessing neck-specific disability, the MSK-HQ offers a unique, holistic assessment of the overall musculoskeletal health burden, covering pain, physical function, sleep, and psychosocial aspects. This makes it particularly useful for patients with multiple MSK complaints or for benchmarking care across different MSK conditions, which is an emerging need in both clinical practice and research.

The translation process highlighted the necessity of nuanced adjustments to preserve conceptual equivalence. For instance, distinguishing “aches” (酸痛) from “pains” (疼痛) addressed a linguistic gap in Chinese, where these terms convey distinct sensory experiences. Similarly, rephrasing “washing” as 洗漱 (wash one’s face and rinse one’s mouth) and “daily routine” as 日常生活安排 (Daily life arrangements) aligned with colloquial expressions, ensuring contextual relevance. Calibrating intensity descriptors (e.g., “extremely” vs. “very”) to 极其 and 非常 further enhanced response accuracy, mitigating potential biases in pain reporting [29]. These adaptations underscore the importance of transcending literal translation to achieve cultural resonance, as emphasized in international PROM guidelines [30].

The absence of floor or ceiling effects suggests that the Simplified Chinese MSK-HQ is well-suited for capturing a broad spectrum of musculoskeletal health in chronic neck pain patients. This contrasts with studies of acute populations, where extreme scores may cluster due to rapid symptom fluctuations [24]. The observed normal score distribution aligns with the chronic nature of our sample, where prolonged disability likely led to stabilized self-perceptions of health.

The high internal consistency (Cronbach’s α = 0.922) and test-retest reliability (ICC = 0.822) are consistent with prior MSK-HQ validations in other languages [11–17]. However, differences in test-retest intervals (7–14 days in this study vs. 3–7 days in others) may explain slight ICC variations, as shorter intervals risk recall bias, while longer periods increase the likelihood of true clinical change. Our interval balanced these concerns, reflecting typical outpatient follow-up schedules in China. It should be noted that the chosen test-retest interval of 7–14 days may influence ICC values when compared to studies using different intervals. Shorter intervals might yield higher ICC due to recall effects, while longer intervals might show lower ICC due to actual clinical changes.

The strong correlations with physical health measures (EQ-5D, CNFDS, SF-36 physical subscales) confirm the tool’s convergent validity, while weaker associations with SF-36 mental subscales (*r* = .120 − .349) support its discriminant validity, aligning with the pattern observed in previous validation studies [10, 12–15]. Construct validity was supported by strong correlations with reference measures. MSK-HQ-C’s correlation with EQ-5D-Total (*r* = .648) is slightly lower than the original English version (*r* = .81) but consistent with the Hungarian (*r* = .788), Arabic (*r* = .711), and German (*r* = .75) versions, indicating cross-cultural convergence. This aligns with the MSK-HQ’s design to prioritize physical and functional impacts of musculoskeletal disorders. Notably, the focus on neck pain, a condition often linked to localized disability rather than global mental health decline, may have accentuated this divergence. Future studies should explore whether similar patterns hold in populations with systemic MSK conditions (e.g., fibromyalgia), where psychological sequelae are more pronounced.

The 2-factor solution identified in EFA (physical function, and emotional/social impact) adds to the evolving evidence that the factor structure of the MSK-HQ may be population- and context-dependent. While it diverges from the original 3-factor model, it finds parallels in other adaptations (e.g., the Norwegian version’s emphasis on physical and psychosocial domains). These differences may reflect variations in cultural conceptualizations of health (e.g., the integration of emotional and social well-being in collectivist societies) as well as the specific clinical characteristics of the study sample (e.g., chronic neck pain patients).

A limitation of this study is that responsiveness of MSK-HQ-C was not assessed, as it was beyond the scope of this cross-sectional validation design. Future longitudinal or interventional studies are needed to evaluate the responsiveness of the MSK-HQ-C. Besides, the two-factor structure identified through EFA on a split-half sample requires confirmation in future studies using Confirmatory Factor Analysis (CFA) in larger, independent, and more diverse samples. Moreover, the sample size was relatively small and are not representative of the mainland Chinese general population. Besides, China is a multinational country, so national cultural gap is also a problem that should be noted. Furthermore, as the validation was conducted exclusively in patients with non-specific chronic neck pain, the findings cannot be directly generalized to other musculoskeletal conditions or community populations without further validation studies.

## Conclusions

Simplified Chinese version of MSK-HQ had sufficient reliability and validity to be used for standard evaluations of patients who suffered from non-specific chronic neck pain in Chinese mainland. However, as the validation was conducted exclusively in patients with non-specific chronic neck pain, the findings cannot be directly generalized to other musculoskeletal conditions without further validation studies. 

## Supplementary Information

Supplementary Material 1.

## Data Availability

The datasets generated and/or analyzed during the current study are not publicly available due to some of the patient’s data regarding individual privacy, and according to the policy of our hospital, the data could not be shared with others without permission, but some are available from the corresponding author on reasonable request.

## Abbreviations

MSK: Musculoskeletal
MSK-HQ: Musculoskeletal Health Questionnaire
MSK-HQ-C: Simplified Chinese version of Musculoskeletal Health Questionnaire
PROMs: Patient-reported Outcome Measures
EQ-5D-5L: EuroQol-5 Dimensions-5 Levels
CNFDS: Copenhagen Neck Function Disability Scale
SF-36: Short Form (36) Health Survey
ICC: Intra-class Correlation Coefficient
KMO: Kaiser-Meyer-Olkin
EFA: Exploratory Factor Analysis
HRQoL: Health-Related Quality of Life
SD: Standard Deviation
CI: Confidence Interval
SPSS: Statistical Package for Social Science
