# Supplementary material for: Reliability and validity of the simplified Chinese version of the Musculoskeletal Health Questionnaire (MSK-HQ-C) in patients with non-specific chronic neck pain: a cross-cultural adaptation and validation study
Source: BMC Psychol. 2026 Mar 13;14:574. doi: 10.1186/s40359-026-04288-w (PMC13097534; doi:10.1186/s40359-026-04288-w)
Supplement: Supplementary file 1 — Supplementary Material 1. [file 40359_2026_4288_MOESM1_ESM.docx]

**Table S1.** Demographic and clinical characteristics of the pilot test participants (n=15).

| **Characteristic** | **Value** |
| --- | --- |
| **Age (years)** |  |
| Mean ± SD | 45.7 ± 8.4 |
| Range | 31 - 63 |
| **Gender, n (%)** |  |
| Male | 7 (46.7%) |
| Female | 8 (53.3%) |
| **Pain Duration (months)** |  |
| Mean ± SD | 13.3 ± 7.7 |
| Range | 4 - 24 |
| **Type of Neck Pain, n (%)** |  |
| Non-specific chronic neck pain | 15 (100%) |
| **Previous Treatments, n (%)** |  |
| Physical therapy | 9 (60.0%) |
| Medication | 11 (73.3%) |
| Acupuncture | 5 (33.3%) |
| None | 2 (13.3%) |

SD: Standard Deviation.

**Table S2.** Correlations of the two MSK-HQ-C factors with criterion measures.

| Scales | Factor 1: Physical Function (r) | Factor 2: Emotional/Social Impact (r) |
| --- | --- | --- |
| **EQ-5D** |  |  |
| Total score | .646^**^ | .615^**^ |
| Health status (VAS) | .662^**^ | .726^**^ |
| **CNFDS** | -.747^**^ | -.732^**^ |
| **SF-36 subscales** |  |  |
| Physical Function | .604^**^ | .549^**^ |
| Role-Physical | .484^**^ | .427^**^ |
| Bodily Pain | .520^**^ | .476^**^ |
| General Health | .502^**^ | .463^**^ |
| Vitality | .260^**^ | .263^**^ |
| Social Function | .213^*^ | .183^*^ |
| Role-Emotional | .115 | .119 |
| Mental Health | .373^**^ | .302^**^ |

*p < .05, **p < .01. EQ-5D: EuroQol-5 dimensions; CNFDS: Copenhagen Neck Functional Disability Scale; SF-36 subscales: PF-Physical Functioning, RP-Role-Physical, BP-Bodily Pain, GH-General Health, VT-Vitality, SF-Social Functioning, RE-Role-Emotional, MH-Mental Health.
